# Effects of *SLC22A2* 808G>T polymorphism and bosutinib concentrations on serum creatinine in patients with chronic myeloid leukemia receiving bosutinib therapy

**DOI:** 10.1038/s41598-021-85757-7

**Published:** 2021-03-18

**Authors:** Maiko Abumiya, Naoto Takahashi, Saori Takahashi, Tomoko Yoshioka, Yoshihiro Kameoka, Masatomo Miura

**Affiliations:** 1 Department of Pharmacy, Akita University Hospital, 1-1-1 Hondo, Akita, 010-8543 Japan; 2 Department of Hematology, Nephrology, and Rheumatology, Akita University Graduate School of Medicine, Akita, Japan; 3 Clinical Research Promotion and Support Center, Akita University Hospital, Akita, Japan

**Keywords:** Cancer, Biomarkers, Medical research, Oncology

## Abstract

The purpose of this study was to investigate the effects of *SLC22A2* 808G>T polymorphism and trough concentrations (C_0_) of bosutinib on serum creatinine in 28 patients taking bosutinib. At 1, 3, 6, 12, 24, and 36 months after administration, analysis of bosutinib C_0_ and creatinine was performed at the same time of day. Significant correlations were observed between bosutinib C_0_ and the change rate of serum creatinine or the estimated glomerular filtration rate (eGFR; *r* = 0.328, *P* < 0.001 and *r* = − 0.315, *P* < 0.001, respectively). These correlations were particularly high in patients having the *SLC22A2* 808G/G genotype (*r* = 0.345 and *r* = − 0.329, respectively); however, in patients having the 808T allele, there were no significant differences. In multivariate analyses, the *SLC22A2* 808G/G genotype, patient age, bosutinib C_0_ and second-line or later bosutinib were independent factors influencing the change rate of creatinine. Bosutinib elevated serum creatinine through organic cation transporter 2 (OCT2). We observed a 20% increase in serum creatinine with a median bosutinib C_0_ of 63.4–73.2 ng/mL. Periodic measurement of serum creatinine after bosutinib therapy is necessary to avoid progression to severe renal dysfunction from simple elevation of creatinine mediated by OCT2 following bosutinib treatment.

## Introduction

Bosutinib is a second-generation tyrosine kinase inhibitor (TKI) that acts as a dual inhibitor of Src and ABL kinases^1,2^. In long-term analyses, patients receiving bosutinib therapy have been reported to show declines in renal function, such as an increase from baseline in serum creatinine values and a decrease in the estimated glomerular filtration rate (eGFR)^3^. The decline in eGFR induced by bosutinib is reported to be independent of the dose of bosutinib^3^, and the mechanisms through which bosutinib induces a decline in renal function are still unclear.

Creatinine is actively secreted from tubular epithelial cells via organic cation transporter 2 (OCT2)^4^. Because many TKIs, such as imatinib and crizotinib, inhibit OCT2 within the range of clinically observed concentrations^5–7^, tubular secretion of creatinine is blocked by these TKIs, resulting in increased creatinine concentrations in serum^8^. In an in vitro study, Omote et al. reported that crizotinib and imatinib may increase serum creatinine values by more than 10% based on renal creatinine clearance and the plasma concentrations of these TKIs^5^. Although bosutinib was not previously evaluated^5,7^, research has suggested that OCT2 may contribute to decreased renal function induced by bosutinib. However, to date, no reports have described the relationships between plasma concentrations of bosutinib and serum creatinine values.

Several single nucleotide polymorphisms (SNPs) in OCT2 (encoded by the *SLC22A2* gene) have been identified. Among them, the SNP rs316019 in exon 4 of the *SLC22A2* gene is an 808G>T transversion that results in an amino acid change from serine to alanine at codon 270^9,10^. The OCT2 transport activity in individuals with the *SLC22A2* 808T allele is significantly lower than that in individuals with the 808G/G genotype^11–13^. Therefore, serum creatinine values in patients with the *SLC22A2* 808T allele tend to be higher than in those with the 808G/G genotype^14,15^.

In the current study, we investigated the relationships between trough plasma concentrations of bosutinib and serum creatinine values and assessed the effects of the *SLC22A2* 808G>T polymorphism in patients with Philadelphia chromosome-positive chronic myeloid leukemia (CML) receiving bosutinib therapy.

## Results

Patient characteristics before bosutinib therapy are listed in Table 1. The mean (± SD) age of patients was 55 ± 16 years, and the mean body weight (± SD) was 64 ± 16 kg. There were no patients with serious renal or hepatic dysfunction before bosutinib therapy. Eight and 20 patients received bosutinib therapy as first-line and second-line or later therapy, respectively (Table 1). The change rates of serum creatinine and eGFR at 1 year after bosutinib therapy were significantly higher in patients receiving second-line or later bosutinib than in those receiving first-line bosutinib (each *P* = 0.009; Table 2). Three patients had diabetes, and 7 patients had hypertension before bosutinib therapy; however, there were no significant differences in the change rates of serum creatinine and eGFR between patients with and without diabetes or hypertension.

**Table 1:** Demographic and clinical characteristics of patients prior to bosutinib therapy.

Female:male	13:15	
Age (years)	55 ± 16	(22–80)
Body weight (kg)	64 ± 16	(44–101)
**Bosutinib therapy**		
First-line:second-line or later	8:20	
**Medical history**		
Diabetes (yes:no)	3:25	
Hypertension (yes:no)	7:21	
**Laboratory test values**		
White blood cells (× 10^3^/mm^3^)	16.0 ± 7	(1–77.7)
Platelets (× 10^4^/mm^3^)	41.5 ± 62.6	(12.1–326)
Aspartate transaminase (IU/L)	24 ± 9	(10–56)
Alanine transaminase (IU/L)	25 ± 16	(7–83)
Serum albumin (g/dL)	4.2 ± 0.4	(3.0–4.8)
Total bilirubin (mg/dL)	0.7 ± 0.5	(0.3–2.7)
Serum creatinine (mg/dL)	0.69 ± 0.19	(0.42–1.04)
eGFR (mL/min/1.73 m^2^)	86.0 ± 19.7	(50.2–122)

Data are presented as the mean ± standard deviation (range) or number (%).

**Table 2:** Comparison of laboratory test data between patients receiving bosutinib therapy at first-line and second-line or later.

Bosutinib therapy	First-line		second-line or later		*P* values
Patient number	8		20		
Diabetes	1		2		1.000
Hypertension	2		5		1.000

Laboratory test	Median	(quartile1–quartile3)	Median	(quartile1–quartile3)	*P* values
**Baseline before bosutinib therapy**					
Serum creatinine (mg/dL)	0.80	(0.67 to 0.89)	0.61	(0.47 to 0.85)	0.075
eGFR (mL/min/1.73 m^2^)	71.6	(65.0 to 91.0)	89.1	(74.1 to 111.5)	0.047
**One year after bosutinib treatment**					
Serum creatinine (mg/dL)	0.83	(0.76 to 0.99)	0.81	(0.62 to 0.93)	0.658
Change rate of serum creatinine (%)	10.0	(5.1 to 15.2)	31.8	(13.0 to 45.3)	0.009
eGFR (mL/min/1.73 m^2^)	61.6	(56.5 to 79.8)	69.3	(50.2 to 83.3)	0.938
Change rate of eGFR (%)	− 11.4	(− 14.7 to − 5.7)	− 26.5	(− 33.9 to − 13.3)	0.009
Bosutinib daily dose (mg)	300	(200 to 400)	400	(300 to 400)	0.577
Bosutinib C_0_ (ng/mL)	59.7	(48.4 to 80.2)	74.8	(46.5 to 104.5)	0.498

*C*_*0*_ trough plasma concentration.

**Figure 1:**
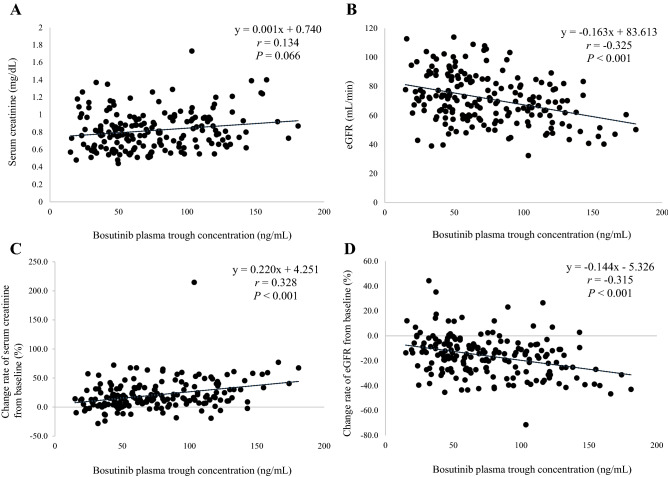
The relationships of bosutinib plasma trough concentrations with (**A**) serum creatinine, (**B**) eGFR, (**C**) change rate of serum creatinine from baseline, and (**D**) change rate of eGFR from baseline.

For all plasma samples collected from 1 to 36 months after bosutinib administration, the relationships of bosutinib C_0_ with serum creatinine, eGFR, or change rates are shown in Fig. 1. There were no significant relationships between bosutinib C_0_ and serum creatinine values at the same time (Fig. 1A). However, a significant correlation between bosutinib C_0_ and eGFR was observed (*r* = − 0.325, *P* < 0.001; Fig. 1B). In addition, significant correlations between bosutinib C_0_ and the change rates of serum creatinine and eGFR were also observed (*r* = 0.328, *P* < 0.001 and *r* = -0.315, *P* < 0.001, respectively; Fig. 1C,D).

**Figure 2:**
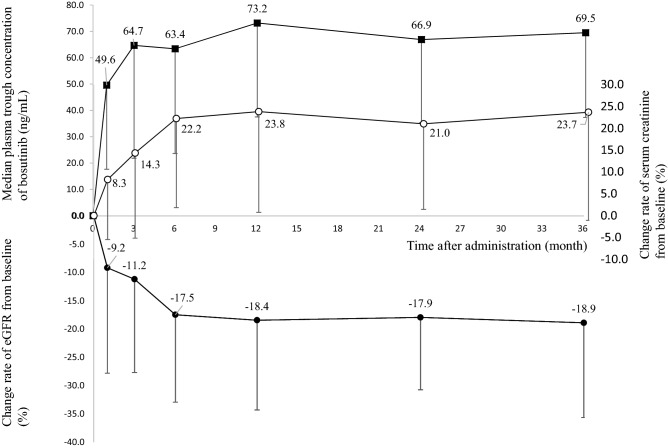
Time course of median plasma trough concentrations of bosutinib (closed squares) and change rates from baseline of serum creatinine (opened circles) and eGFR (closed circles) after bosutinib administration.

The transition of bosutinib C_0_ and the change rates from baseline of serum creatinine and eGFR after bosutinib administration are shown in Fig. 2. The slopes for change rates of serum creatinine and eGFR were large within 6 months after bosutinib administration.

Twenty-four and four patients had the *SLC22A2* 808G/G genotype and 808T allele, respectively (Table 3). The change rates of serum creatinine and eGFR at 1 year after bosutinib therapy were higher in patients having the *SLC22A2* 808G/G genotype than in those having the 808T allele; however, the difference was not significant. Significant correlations between bosutinib C_0_ and the change rates of serum creatinine or eGFR in patients having the *SLC22A2* 808G/G genotype were observed (*r* = 0.345, *P* < 0.001, and *r* = − 0.329, *P* < 0.001, respectively); however, in patients having the *SLC22A2* 808T allele, there were no significant correlations between bosutinib C_0_ and the change rates of serum creatinine or eGFR.

**Table 3:** Comparison of laboratory test data after bosutinib administration between patients with the *SLC22A2*808G/G genotype and 808T allele.

*SLC22A2* 808G>T polymorphism	*SLC22A2* 808G/G		*SLC22A2* 808G/T + T/T		*P* values
Patient number	24		4		
First-line:second-line or later	6:18		2:2		0.555
Diabetes	3		0		1.000
Hypertension	6		1		1.000

Laboratory test	Median	(quartile1–quartile3)	Median	(quartile1–quartile3)	*P* values
**Baseline before bosutinib therapy**					
Serum creatinine (mg/dL)	0.66	(0.53 to 0.85)	0.79	(0.55 to 0.88)	0.577
eGFR (mL/min/1.73 m^2^)	80.8	(71.5 to 105.0)	86.9	(58.6 to 93.9)	0.743
**One year after bosutinib treatment**					
Serum creatinine (mg/dL)	0.84	(0.67 to 0.99)	0.78	(0.64 to 0.82)	0.339
Change rate of serum creatinine (%)	21.6	(8.8 to 43.8)	3.3	(− 10.4 to 19.2)	0.088
eGFR (mL/min/1.73 m^2^)	68.2	(53.9 to 75.1)	76.7	(60.5 to 90.4)	0.306
Change rate of eGFR (%)	− 19.6	(− 33.1 to − 10.4)	− 2.2	(− 17.8 to 11.9)	0.088
Bosutinib daily dose (mg)	400	(300 to 400)	350	(225 to 400)	0.537
Bosutinib C_0_ (ng/mL)	74.8	(48.0 to 97.7)	49.4	(46.4 to 73.2)	0.290

Correlation coefficient with bosutinib C_0_ for all samples during the 3 years after bosutinib treatment	*r*	*P* values	*r*	*P* values	
Change rate of serum creatinine (%)	0.345	< 0.001	− 0.010	0.961	
Change rate of eGFR (%)	− 0.329	< 0.001	0.004	0.984	

*C*_*0*_ trough plasma concentration.

Stepwise selection multiple linear regression analysis of explanatory variables for the change rate of serum creatinine is shown in Table 4. The *SLC22A2* 808G/G genotype, patient age, bosutinib C_0_ and second-line or later bosutinib therapy were independent factors influencing the change rate of serum creatinine (*P* = 0.003, < 0.001, = 0.008, and < 0.001, respectively); however, the determination coefficient for the change rate of serum creatinine was 0.333.

**Table 4:** Stepwise multiple regression analysis of explanatory variables for the change rate of serum creatinine.

Explanatory variable	Slope	SE	SRC	*P* value	*R*^2^
*SLC22A2* 808G>T polymorphism (T allele = 1)	− 13.689	4.527	− 0.189	0.003	0.333
Age (years)	0.441	0.107	0.259	< 0.001	
Bosutinib C_0_ (ng/mL)	0.118	0.044	0.172	0.008	
Previous TKI therapy (second-line or later bosutinib = 1)	18.442	3.386	0.340	< 0.001	
Intercept	− 23.113	6.582			

*SE* standard error, *SRC* standardized regression coefficient, *C*_*0*_ trough plasma concentration, *TKI* tyrosine kinase inhibitor.

## Discussion

Changes in serum creatinine and eGFR in CML patients after bosutinib administration were significantly correlated with bosutinib C_0_ values. In particular, the correlation coefficient between changes in serum creatinine or eGFR and bosutinib C_0_ was higher in patients with the *SLC22A2* 808G/G genotype than in the total patient cohort in this study. Because patients with the *SLC22A2* 808T allele have lower OCT2 transport activity^11–13^, creatinine values in serum always tends to be high; hence, the change rate of serum creatinine following administration of bosutinib in patients with the *SLC22A2* 808T allele seem low. In contrast, significant elevation of serum creatinine values was observed in patients with the *SLC22A2* 808G/G genotype. These findings suggested that bosutinib elevated serum creatinine by inhibiting OCT2-dependent transport of creatinine. In addition, multivariate analysis showed that aging was related to elevation of serum creatinine values. To date, a higher bosutinib C_0_ of more than 91.0 ng/ mL has been reported to be associated with an onset of adverse events, such as delayed diarrhea and liver dysfunction^16^. Therefore, in elderly patients with the *SLC22A2* 808G/G genotype, administration of bosutinib at doses that would yield high bosutinib C_0_ should be avoided.

In a phase 3 trial of bosutinib for CML treatment (the BFORE trial), the median C_0_ of bosutinib after administration of an initial dose of bosutinib 400 mg once daily was 55.75 ng/mL^17^, whereas that after administration of an initial dose of bosutinib 500 mg once daily in the Bosutinib Efficacy and Safety in Newly Diagnosed CML (BELA) trial was 67.51 ng/mL^17^. In pooled data from the two trials, the median C_0_ of bosutinib was reported to be 61.29 ng/mL^17^. Our previous study showed that the median bosutinib C_0_ in the maintenance phase was 63 ng/mL^16^. Therefore, the target bosutinib C_0_ to obtain better responses and decrease the risk of adverse events was approximately 62 ng/mL^18^. In the current study, the median bosutinib C_0_ from 3 to 36 months after administration of bosutinib ranged from 63.4 to 73.2 ng/mL. Similar to imatinib^5,7^, an increase in serum creatinine by bosutinib also seemed to occur via inhibition of OCT2; however, bosutinib appeared to inhibit OCT2, even at a plasma concentration of around 62 ng/mL. In particular, the slopes of the change rates of serum creatinine and eGFR during the 6 months after administration of bosutinib were large; accordingly, an increase in serum creatinine of approximately 20% by 6 months after administration of bosutinib was observed. It is possible that the inhibitory effects of bosutinib for OCT2 did not appear to be strong because the time required for elevation of serum creatinine was long. Careful monitoring of serum creatinine is necessary during the 6 months after beginning bosutinib therapy. Consequently, we observed a 20% increase in serum creatinine by transition of the bosutinib C_0_ to 60–70 ng/mL during the 3 years after bosutinib therapy. For maintenance of the approximately 20% increase in serum creatinine, dose adjustment according to the target bosutinib C_0_ of approximately 62 ng/mL may be necessary. In the current study, patients having a treatment history of imatinib, nilotinib, or dasatinib also showed elevation of serum creatinine values after bosutinib therapy. This finding was similar to the results of a previous report^3^. In patients with CML receiving second-line or later bosutinib, a dose escalation regimen based on the target bosutinib C_0_ of approximately 62 ng/mL may be effective^16^.

Analysis of the *SLC22A2* 808G>T polymorphism before bosutinib administration could predict the increase in serum creatinine after the beginning of treatment. However, because the genotype frequency of *SLC22A2* 808G/G in the Japanese population is 89.0%^10,14,15^, serum creatinine values in many patients are altered by administration of bosutinib. In the current study, the genotype frequency of *SLC22A2* 808G/G was 85.7%, and allele frequencies for the different analyzed loci were at Hardy–Weinberg equilibrium. Therefore, periodic measurement of serum creatinine and bosutinib C_0_ after bosutinib therapy is more important than analysis of the *SLC22A2* 808G>T polymorphism before bosutinib administration. In the current study, the daily dose of bosutinib was not a predictor of elevation of serum creatinine. This finding was similar to the results of a previous report^3^. The plasma concentrations of bosutinib do not show dose dependence, but do exhibit saturation for doses above 300 mg/day^1,19,20^. This phenomenon may explain why the increase in serum creatinine induced by bosutinib was not dose-dependent. Although the increase in serum creatinine of approximately 20% within 6 months after administration of bosutinib was not clinically significant, periodic measurement of serum creatinine is necessary to avoid progression to severe renal dysfunction from simple elevation of serum creatinine values mediated by OCT2 following bosutinib treatment. Increases in serum creatinine of more than 20% may be related to other causes, such as vascular occlusive events, and are a very important adverse event, particularly in elderly patients with CML.

In multivariate analyses, the proportion of the 4 explanatory variables for the increase in serum creatinine, that is, *SLC22A2* 808G/G genotype, patient age, bosutinib C_0_, and history of previous TKI therapy, was 33.3%, which was relatively low. Several factors may have affected this result. For example, after the beginning of bosutinib treatment, OCT2-mediated creatinine transport may be inhibited by the addition of therapeutic agents for other disorders. Alternatively, renal function in patients with a history of diabetes or hypertension may be reduced. However, we were not able to clarify this reason in the current study. In addition, the results from the current study were obtained by analysis of data during the 36 months after bosutinib administration, and the results of long-term use of bosutinib are unclear. Therefore, our current findings should be interpreted within the context of the study limitations. Additional studies may be necessary.

## Conclusion

Changes in serum creatinine after bosutinib therapy were significantly correlated with bosutinib C_0_ values. An increase in serum creatinine of approximately 20% by transition of the bosutinib C_0_ to 60–70 ng/mL at the maintenance phase was confirmed. In particular, in patients with the *SLC22A2* 808G/G genotype, significant elevation of serum creatinine by bosutinib was detected. After beginning bosutinib therapy, we may need to confirm the bosutinib C_0_ and perform periodic measurement of serum creatinine.

## Methods

### Patients and protocols

Twenty-eight Japanese patients with Philadelphia chromosome-positive CML (13 women and 15 men) taking bosutinib (Bosulif; Pfizer, Tokyo, Japan), who were treated at Akita University Hospital from June 2010 through June 2020 were prospectively enrolled in the study. Seventeen patients in this study had participated in our previous studies^16^. The demographic and clinical characteristics of the patients prior of bosutinib therapy are listed in Table 1. The study was conducted according to the principles of the Declaration of Helsinki. The study protocol was approved by the Ethics Committee of Akita University School of Medicine (approval number: 1140), and all patients provided written informed consent for participation in the study.

### Sample collection

Bosutinib was orally administered once daily in the morning. Reductions in bosutinib daily dosage were carried out based on the grade of each side effect. At 1, 3, 6, 12, 24, and 36 months after bosutinib administration, whole blood samples were collected by venipuncture at 24 ± 2 h after administration (trough plasma concentration, C_0_). Plasma was isolated by centrifugation at 1900×*g* for 15 min and was stored at − 40 °C until analysis. Plasma concentrations of bosutinib were measured by high-performance liquid chromatography^16^. Analysis of bosutinib C_0_ and serum creatinine was performed at the same time of day. Twenty-eight patients who completed these evaluations were retrospectively analyzed.

### Identification of genotypes

DNA was extracted from peripheral blood samples using a QIAamp Blood Kit (Qiagen, Hilden, Germany) and was stored at − 80 °C until analysis. Genotyping procedures identifying the G and T alleles of *SLC22A2* were performed using polymerase chain reaction-restriction fragment length polymorphism (PCR-RFLP), as described by Wang et al*.*^12^.

### Statistical analyses

The clinical characteristics of patients were expressed as numbers or mean values ± standard deviations (SDs) and ranges. The eGFR was calculated using the following formulas: eGFR = 194 × serum creatinine concentration (mg/dL)^−1.094^ × age^−0.287^ (× 0.739 for women)^21^. The change rate in laboratory data = (after bosutinib therapy – before bosutinib therapy)/before bosutinib therapy.

The Kolmogorov–Smirnov test was applied to assess the distribution in each dataset. Spearman’s rank correlation coefficient tests were used to assess correlations of bosutinib C_0_ with serum creatinine, eGFR, or the change rates, and all results were expressed as correlation coefficients (*r*). The bosutinib C_0_ for each genotype of *SLC22A2* 808G>T was expressed as the median (quartile 1 and 3), and Mann–Whitney *U* tests were used to determine differences between genotype groups.

The effects of factors in univariate analysis were evaluated using stepwise multiple linear regression analysis. For each patient, dummy variables (1 and 0) were used to replace the genotypes of *SLC22A2* 808G>T. Differences or correlations with *P* values of less than 0.05 were considered statistically significant. Statistical analysis was performed using SPSS 20.0 for Windows (SPSS IBM Japan Inc., Tokyo, Japan).

### Ethics approval

Approval number: 1140.

### Consent to participate

Informed consent: signed informed consent was obtained from all patients.

### Research involving human participants

This study was performed in accordance with the ethical standards of the Declaration of Helsinki and its subsequent amendments.

## Data Availability

All data generated or analysed during this study are included in this published article.
